# Co-Deposition of a Hydrogel/Calcium Phosphate Hybrid Layer on 3D Printed Poly(Lactic Acid) Scaffolds via Dip Coating: Towards Automated Biomaterials Fabrication

**DOI:** 10.3390/polym10030275

**Published:** 2018-03-07

**Authors:** Matthias Schneider, Christina Günter, Andreas Taubert

**Affiliations:** 1 Institute of Chemistry, University of Potsdam, D-14476 Potsdam, Germany; mattschn@uni-potsdam.de; 2 Institute of Earth and Environmental Sciences, University of Potsdam, D-14476 Potsdam, Germany; guenter@geo.uni-potsdam.de

**Keywords:** 3D printing, dip-coating, poly(lactic acid), PLA, calcium phosphate, gelatin, chitosan, hydrogel, calcium phosphate hybrid material, biomaterials

## Abstract

The article describes the surface modification of 3D printed poly(lactic acid) (PLA) scaffolds with calcium phosphate (CP)/gelatin and CP/chitosan hybrid coating layers. The presence of gelatin or chitosan significantly enhances CP co-deposition and adhesion of the mineral layer on the PLA scaffolds. The hydrogel/CP coating layers are fairly thick and the mineral is a mixture of brushite, octacalcium phosphate, and hydroxyapatite. Mineral formation is uniform throughout the printed architectures and all steps (printing, hydrogel deposition, and mineralization) are in principle amenable to automatization. Overall, the process reported here therefore has a high application potential for the controlled synthesis of biomimetic coatings on polymeric biomaterials.

## 1. Introduction

New polymer/inorganic bone repair materials based on calcium phosphate (CP) are a major topic in current biomaterials research [1]. One of the most popular scaffold materials is poly(lactic acid) (PLA), a biocompatible and biodegradable polyester [2]. The combination of PLA with CP is an excellent starting point for the development of synthetic bone materials [3,4,5,6,7]. One of the issues when constructing such materials, however, is the interface between the synthetic material and the biological environment [4]. 

Hydrogels can provide a water-rich and soft contact area between a synthetic polymer or hybrid material of an implant and the surrounding biological tissue [8]. Hydrogels can provide an ideal contact medium for cell attachment and growth. They enable the (re)generation of bone and similar tissue [9]. Moreover, hydrogel layers can also be loaded with antibiotics or growth factors to enhance cell growth and/or peptides or proteins fostering mineral deposition [7,10]. Finally, hydrogel contact layers can be pre-treated with calcium phosphate (CP) or calcium carbonate (CC) to provide an initial mineral reservoir that can be used by the organism for enhanced mineral deposition [1].

Nowadays, many methods for the controlled deposition of CP exist [8,11,12]. A particularly popular method is the soaking of material in simulated body fluid (SBF) as developed by Kokubo et al. [3,13]. A further method by Taguchi et al. [3,14] is based on soaking the scaffold in a calcium-containing solution followed by soaking in a hydrogen phosphate-containing solution. Repeated application of these soaking steps yields CP layers with a controlled composition and architecture on a substrate. This in turn provides access to CP layers with controllable and adaptable properties, which can be adjusted to a desired application, for example in bone repair. 

To improve the mechanical properties of hydrogel-based materials, a more rigid polymer can be used as an underlying scaffold onto which the hydrogel layer is deposited. PLA has attracted tremendous attention as a biomaterial because it is biocompatible and biodegradable [2,15]. Furthermore, it is thermoplastic and can easily be processed and molded in a thermal process. Due to these properties, PLA has increasingly been used in 3D printing, especially in fused deposition modelling (FDM) [16]. FDM is an effective tool for shaping PLA and has therefore been used extensively for the fabrication of scaffolds and materials with intended application in the biomaterials field [3,6].

The combination of biodegradable, yet mechanically robust, polymer scaffolds like PLA and a mineral component like CP with an adjustable hydrogel contact layer is therefore an interesting approach towards complex 3D polymer/inorganic hybrid materials for bone repair. As a result, strategies to modify PLA surfaces with hydrogels for enhanced mineralization is a highly relevant topic for advanced biomaterials development. The current article provides an approach towards such materials using a simple, reproducible, and efficient method that is adaptable to many different materials and material geometries. The current study is inspired by the work of Meng et al. [3] and focuses on the surface modification of 3D printed PLA scaffolds with various hydrogel layers and the concurrent deposition of a CP/hydrogel hybrid layer, which should ultimately support the interaction of the final material with the surrounding biological environment, e.g., for bone formation. 

## 2. Materials and Methods

### 2.1. Materials

Poly(lactic acid) (PLA) filament (1.75 mm diameter, nature, Renkforce (Conrad Electronic SE), Hirschau, Germany), chitosan (≥75% deacetylated, Sigma-Aldrich, St. Louis, MO, USA), glutaraldehyde solution (GA, 25% in water, Sigma-Aldrich), toluene (≥99.7%, Sigma-Aldrich), gelatin (food quality, RUF Lebensmittelwerk KG, Quakenbrück, Germany), (3-Aminopropyl) triethoxysilane (APTES, 98%, Alfa-Aesar, Haverhill, MA, USA), sodium hydroxide (technical, Carl Roth, Karlsruhe, Germany), acetic acid (100%, Carl Roth), calcium nitrate tetrahydrate (≥99%, Carl Roth), disodium hydrogen phosphate (≥98.0%, Fluka, Sigma-Aldrich, Karlsruhe, Germany), and ethanol (abs., 100%, VWR, Radnor, PA, USA) were used as received.

### 2.2. 3D Printing of PLA Scaffolds

PLA scaffolds were printed via fused deposition modelling (FDM) on a CTC 3D printer. Layer resolution was 0.1 mm in z and 0.32 mm in x and y with an 0.4 mm nozzle. Print temperature was 220 °C, bed temperature was 80 °C, and nominal print speed was about 2400 mm∙min^−1^. 

**Figure 1:**
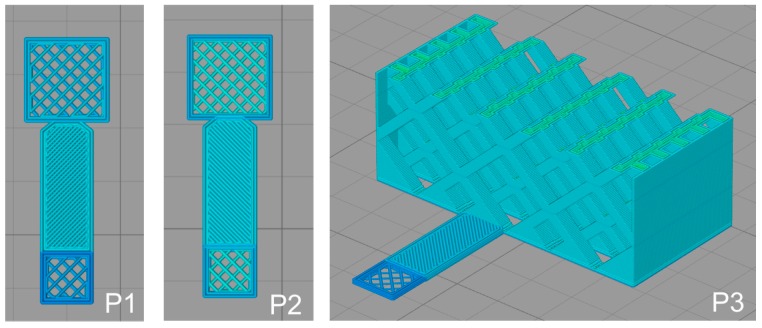
3D models of P1, P2, and P3 scaffolds.

3D models were created using the browser-based editor tinkercad (www.tinkercad.com). For slicing and control of the 3D printer simplify3d (www.simplify3d.com) was used. Three scaffold types were printed, P1–P3 see Figure 1. P1 and P2 are square grid plates with 8 mm edges, 30% infill, and a thickness of 0.3 mm (P1) or 0.6 mm (P2). All P1 and P2 scaffolds have a holding element attached to one side of the square; the dimensions are 17 × 4 mm^2^. P3 scaffolds are rectangular cuboids of 32 × 15 × 12 mm^3^ containing macroscopic pores of about 3.7 mm in diameter. The STL files with the 3D models and the gcode files for the printer are available upon request. 

### 2.3. Modification of PLA Scaffolds

3D printed PLA scaffolds were soaked in 0.5 M aqueous NaOH for 18 h, rinsed with ultrapure water, and dried at 40 °C overnight. P1 and P2 scaffolds were treated with 10 mL of NaOH and P3 scaffolds with 40 mL [17]. 

For subsequent silanization of the scaffolds, 1% of (3-aminopropyl)triethoxysilane (APTES) in toluene was used [18,19]. The P1 and P2 scaffolds were treated with 10 mL of the solution for 23 h and the P3 scaffolds were treated with 40 mL for 23 h. All samples were rinsed with toluene, ethanol, ultrapure water, and then dried at 40 °C overnight. An alternative silanization procedure (to avoid toluene) used a solution of 10% APTES in ethanol following the same procedure as described above, except the washing with toulene.

### 2.4. Combined Hydrogel Deposition and Calcium Phosphate Mineralization

Concurrent hydrogel formation and CP deposition was achieved via a dip coating method adapted and modified from Taguchi et al. [14] and Meng et al. [3]. Initially, two solutions were prepared. The first solution was an aqueous solution of chitosan/calcium nitrate tetrahydrate or gelatin/calcium nitrate tetrahydrate with calcium concentrations between 0.05 and 0.20 mol∙L^−1^. Gelatin or chitosan concentrations were between 0.1% and 1.0% (*w*/*w*) and chitosan was previously dissolved in 1% aqueous acetic acid. The second solution was an aqueous disodium hydrogen phosphate solution with concentrations between 0.05 and 0.20 mol∙L^−1^ containing glutaraldehyde (GA) with concentrations between 0.1% and 1.0% (*w*/*w*). 

**Figure 2:**
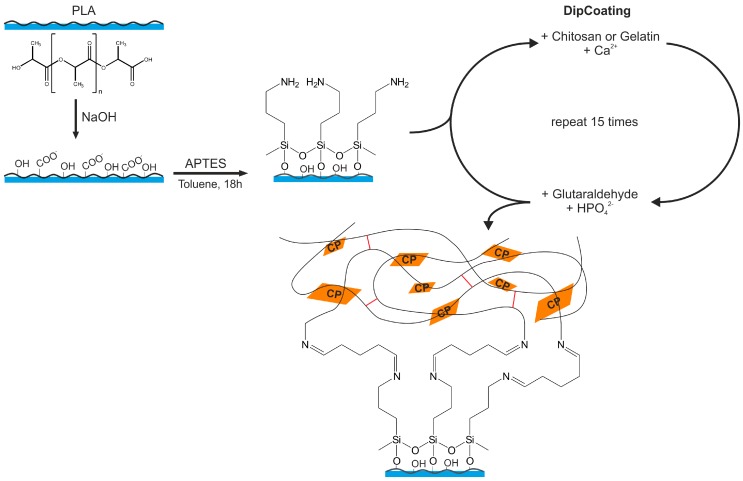
Preparation of the coated PLA scaffolds via repeated alternating dip coating. Red lines indicate glutaraldehyde crosslinks between the polymer chains.

Using a home-built dip coater with two dipping positions, the scaffolds were immersed in the first solution for 5 min, then immersed in the second solution for another 5 min. This automated cycle was repeated 15 times. For analysis, all samples were dried at 40 °C overnight. Figure 2 summarizes the synthesis procedure. 

### 2.5. Characterization 

Attenuated total reflectance infrared (ATR-IR) spectroscopy was done on a Thermo Nicolet Nexus FT-IR-spectrometer (Thermo Fisher Scientific, Waltham, MA, USA) between 400 and 4000 cm^−1^ with a step width of 4 cm^−1^ and 64 scans using a diamond ATR bridge.

Static contact angle (CA) measurements were done on a KSV CAM100 (KSV Instruments, Helsinki, Finland). Samples were investigated as obtained after drying. Ultrapure water was used as testing liquid (ca. 2 µL per drop). The measurements were done on three different locations of the holding element. Each measurement was done in duplicate. 

Atomic force microscopy (AFM) was done on a FlexAFM (Nanosurf, Liestal, Switzerland) with Nanosurf C3000 (Nanosurf) in air using n-type silicon tips with a typical resonance frequency of 160 kHz and a typical force constant of 5 N/m. Measurements were done in tapping mode. 

Scanning electron microscopy (SEM) was done on a JEOL JSM-6510 (Jeol, Tokio, Japan) with a tungsten filament operated at 5 or 10 kV. Energy-dispersive X-ray spectroscopy (EDXS) was done on an Oxford Instruments INCAx-act detector (Oxford Instruments, Uedem, Germany) using a 15 kV acceleration voltage in the SEM. Samples for SEM and EDXS were carbon-coated using a POLARON CC7650 carbon coater.

Differential scanning calorimetry (DSC) was done on a Netzsch Polyma 214 (Netzsch, Selb, Germany) between 25 and 250 °C with a heating rate of 10 K∙min^−1^. Typically 8.8 mg of material were used for the measurement in Al crucibles.

Thermogravimetric analysis (TGA) was done on a Netzsch TG 209F1 Libra (Netzsch, Selb, Germany) with Pt crucibles between 30 and 1000 °C with a heating rate of 10 K∙min^−1^ in N_2_. Between 6 and 18 mg of material were used per measurement.

Powder X-ray diffraction (XRD) was done on a PANalytical Empyrean (Malvern Panalytical B.V., Almelo, The Netherlands) with a Cu K_α_ source and a theta-theta geometry. Total scan time was 21 min over a 2θ range from 4° to 70° using a step size of 0.0131°. 

## 3. Results

### 3.1. Thermal Analysis of PLA Filaments

**Figure A1:**
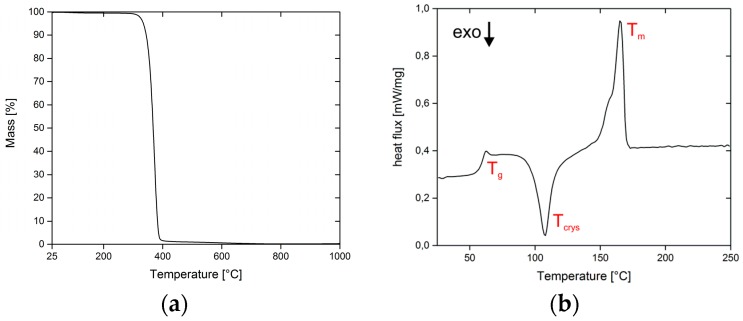
TGA curve (**a**); and DSC curve (**b**) of PLA. *T*_g_ is the glass transition temperature, *T*_crys_ is the cold crystallization, and *T*_m_ is the melting temperature.

To set the 3D printing parameters, the PLA filament was studied with thermogravimetric analysis (TGA) and differential scanning calorimetry (DSC, Figure A1, Appendix A). DSC data show a glass transition at 57.4 °C, a cold crystallization at 97.6 °C, and a melting transition at 156.6 °C. TGA shows a complete one-step degradation between 320 and 373 °C. As a result of this thermal behavior, the 3D printing conditions were chosen as detailed in the experimental part. 

### 3.2. Calcium Phosphate Mineralization on Unmodified PLA Scaffolds

First (control) experiments were done without any previous modification of the PLA scaffolds. Mineral coating was achieved via dip coating (Section 2.4); the experimental parameters and results are summarized in Table 1. The scaffolds show a white coating. This coating, however, is only weakly bound to the surface. Drying is sufficient for the coating to fall off the PLA scaffold. 

### 3.3. Surface Modification of PLA Scaffolds

As direct coating of the PLA scaffolds does not lead to a stable coating, the 3D printed PLA scaffolds were first treated with aqueous NaOH to activate the surface, that is, to cleave some of the ester bonds and to provide –COOH and –OH groups to enable attachment of the silane layers (see Figure 2 and text below). Before and after NaOH treatment, the PLA scaffolds were weighed and all samples show a weight loss with NaOH treatment, Table 2. 

Contact angle (CA) measurements also show that the NaOH treatment changes the PLA surface. While as-printed scaffolds have a CA of 78.0° ± 17.7°, the CA increases to 102.9° ± 9.2° after the treatment. Both the contact angle changes and the weight losses are independent of the scaffold geometry. 

**Figure 3:**
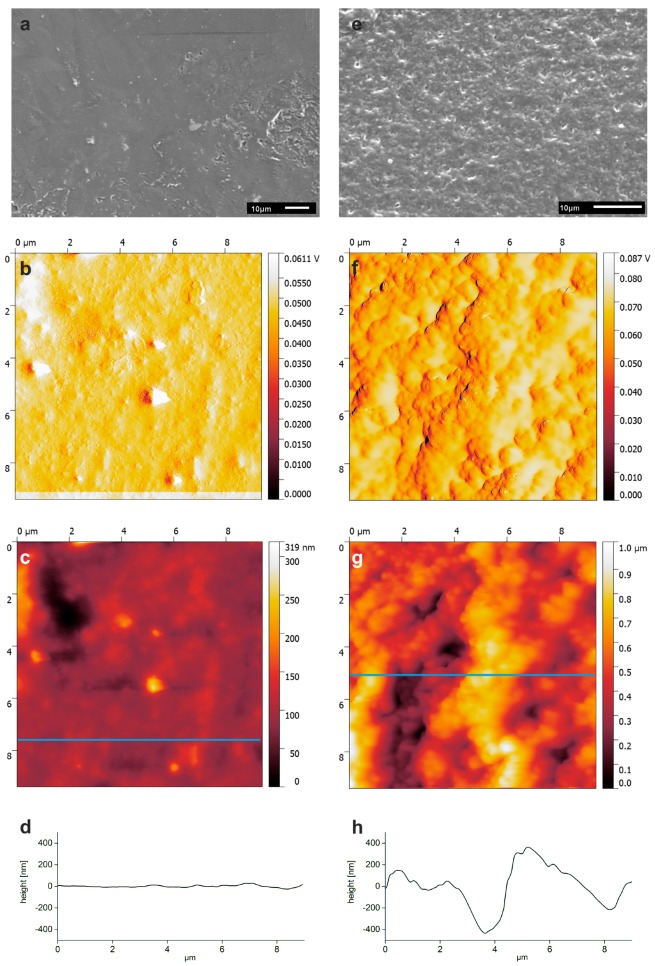
SEM and AFM data on as-printed (**a**–**d**) and NaOH-treated (**e**–**h**) PLA scaffolds. (**a**,**e**) SEM images; (**b**,**f**) AFM amplitude image; and (**c**,**g**) AFM topography image with corresponding height profiles. Blue lines in the images indicate position of the height profiles shown in (**d**,**h**).

Figure 3 shows corresponding scanning electron microscopy (SEM) and atomic force microscopy (AFM) data from the as-printed and NaOH-treated samples. SEM clearly shows that the NaOH treatment has a significant effect on the PLA surface. Before treatment, the surface is relatively smooth and only exhibits a few distinct features with sizes around 0.3 to 0.6 µm and a maximum feature height of up to 300 nm.

After NaOH treatment, the surface is more rugged; this is visible as brighter and darker areas in the SEM images. AFM confirms this observation and detects height differences of up to 1 µm. Surface roughnesses calculated from the entire AFM topography images also confirm the effect of the NaOH treatment; the root mean square (RMS) roughness increases from 48 ± 24 nm (untreated) to 175 ± 63 nm upon NaOH treatment. 

**Figure 4:**
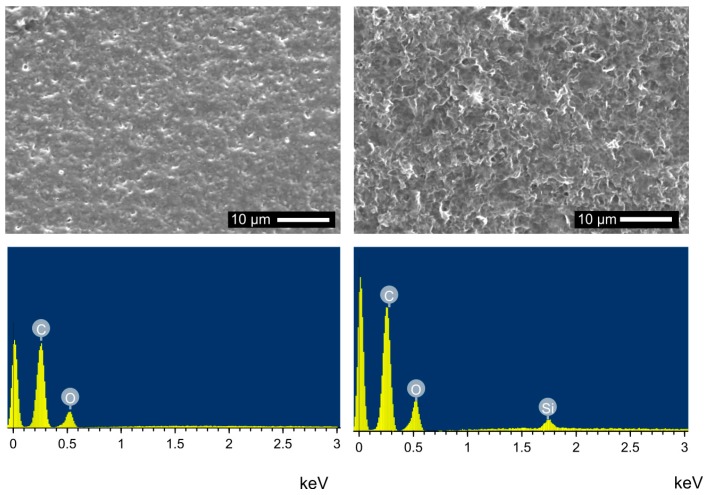
SEM images and EDXS spectra of NaOH-treated PLA before (**left**) and after (**right**) modification with APTES.

After the NaOH treatment, the scaffolds were treated with (3-aminopropyl)triethoxysilane (APTES) to provide binding sites for the subsequent gelatin or chitosan attachment to the PLA surface. Figure 4 shows representative SEM images of a surface after NaOH treatment and after subsequent silanization. The changes in surface appearance are not as significant as after the treatment with NaOH.

Successful silanization is demonstrated by energy dispersive X-ray spectroscopy (EDXS). All EDX spectra of samples after APTES treatment show the presence of silicon. Prior to silanization no silicon is detected in the samples. The oxygen signal stems from the silanes (or SiO_2_ formed upon silane hydrolysis) and the PLA. The EDX spectra also show a shoulder at about 0.4 keV; this shoulder may indicate the presence of nitrogen, but this assignment is difficult. We assign the low signal intensity of the N signal to: (i) an overlap with the O signal; and (ii) a much lower N than O concentration in the samples: the silanization alone introduces three O atoms for every N atom; moreover, PLA contains two O atoms per repeat unit but no N. Moreover, the amount of PLA in the system is much higher than the amount of APTES; as such, the N signal logically must be very weak. 

Finally, the contact angle of the surfaces does not change after silanization (102.2° ± 8.1° after silanization vs. 102.9° ± 9.2° before silanization). As the silane concentration is rather low, no IR signals indicative of silane modification are observed.

### 3.4. Hydrogel-Calcium Phosphate Composites

The text will initially present the results obtained from the gelatin-based hybrid layers and then discuss the analogous chitosan-based materials; to evaluate the deposition conditions, most of the experiments were done on scaffold P2. To produce the CP/hydrogel hybrid layers, the APTES-treated PLA scaffolds were first immersed in a gelatin- or chitosan-containing aqueous Ca(NO_3_)_2_ solution and then in a glutaraldehyde (GA) containing aqueous Na_2_HPO_4_ solution for 5 min each. This cycle was repeated 15 times. Figure 2 illustrates the procedure.

For fabrication of the gelatin-based composite scaffolds, varying concentrations of gelatin, Ca(NO_3_)_2_, Na_2_HPO_4_, and GA were used. The salt concentration has a direct influence on the amount of CP precipitated on the scaffolds. Generally, higher salt concentrations lead to higher mineral yield. However, salt concentrations above 0.3 mol∙L^−1^ lead to very fast mineralization. This then produces samples with uneven and heterogeneous mineral/hydrogel coatings. In contrast, concentrations below 0.05 mol∙L^−1^ lead to samples without significant mineral content after 15 mineralization cycles. Overall, salt concentrations between 0.05 and 0.3 mol∙L^−1^ thus provide access to materials with homogeneous mineral/hydrogel hybrid layers on the PLA scaffolds. 

Besides the calcium and the phosphate concentration, also the polymer concentration affects the mineral formation process. Higher polymer concentrations inhibit CP mineralization—for example, gelatin concentrations higher than 1.0% produce hydrogel layers on the PLA, but without significant CP fractions. Table 3 summarizes the results from the mineralization experiments with the gelatin-based hydrogel coatings.

**Figure 5:**
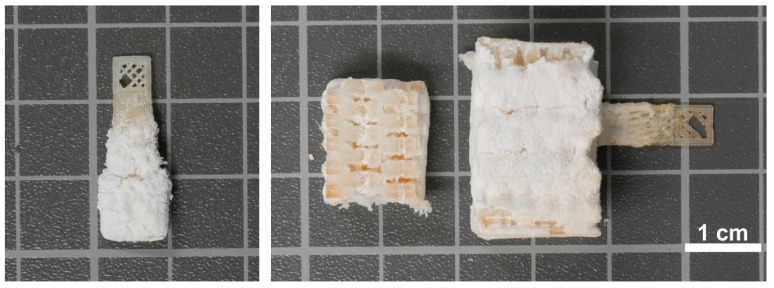
Mineral-coated samples: scaffold type P2 (sample G03 (**left**)) and scaffold type P3 (sample G07 (**center** and **right**)). Sample shown in the center is cut open to show the inner section of the scaffold. The scale bar applies to all images.

After 15 cycles of dip-coating/mineralization, the samples have a thick white coating (Figure 5). The coatings are rather homogeneous and appear firmly connected to the underlying PLA substrate (in contrast to the control PLA samples, see Table 1). Moreover, drying does not destabilize the films as no flaking-off or other degradation of the mineral layers is observed. Some samples do show cracks in the coating after drying, but also these coatings remain intact and do not fall off the PLA surface. Cross-sectional views of samples that are cut open do however show that there is only very little mineral deposition in the inside of the true 3D scaffolds P3. The hybrid layer is about 0.5 mm thick; this thickness is however difficult to determine accurately due to some thickness variation. Furthermore, there are some holes in the mineral coating on the surface, likely due to insufficient mineral deposition. In contrast, the flat P1 and P2 samples also show significant mineralization inside the holes of the mesh structures.

**Figure 6:**
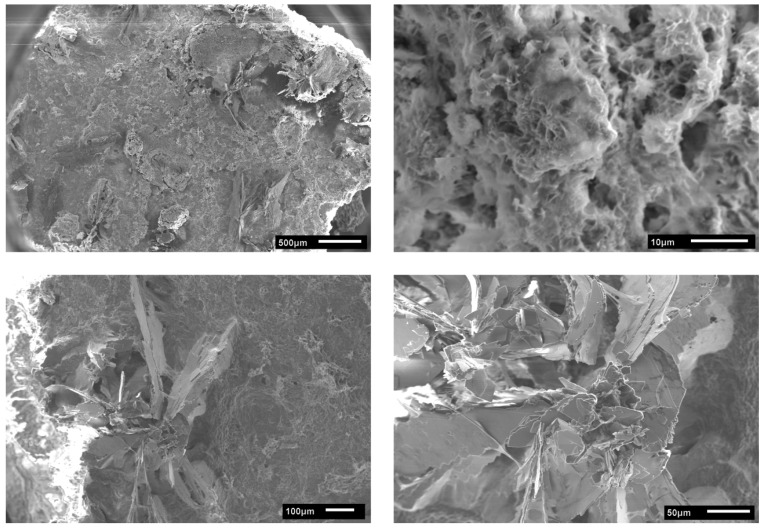
SEM images of gelatin/calcium phosphate hybrid layers on PLA. All images are from the same sample (G01, different sample locations with different magnifications).

SEM (Figure 6) shows a rather homogeneous structure of the mineral/hydrogel hybrid layers with a rough surface morphology. The particle morphologies are quite diverse, ranging from small spherical to rod- or flake-like particles. The largest fraction of the coating is composed of small particles forming a dense thick coating and often show rather complex, sponge-like architectures. Other regions consist of larger, plate-like features that are highly aggregated and intertwined. Due to the rather compact architecture, the determination of particle sizes or size distributions is difficult. SEM however clearly shows that the larger flake-like particles are on the order of a few tens of micrometers. Some locations also show densely packed spherical objects or interconnected needles. 

Complementary EDXS experiments (Table 4) show that there is a difference in the chemical composition of the plates vs. the other morphologies. The plates have a Ca/P ratio of about 1.0; this is indicative of dicalcium phosphate dihydrate (brushite, DCPD) (it must however be noted here that the EDXS experiment also picks up signals form the material underneath the plates—these data are therefore only qualitative) [11]. The less well-ordered regions consisting of the spherical and rod-like particles have a Ca/P ratio of around 1.4. This suggests the presence of calcium-deficient hydroxyapatite (HAP) or octacalcium phosphate (OCP), among others [11]. Moreover, in contrast to the plate-like features, these regions also contain some Na.

**Figure 7:**
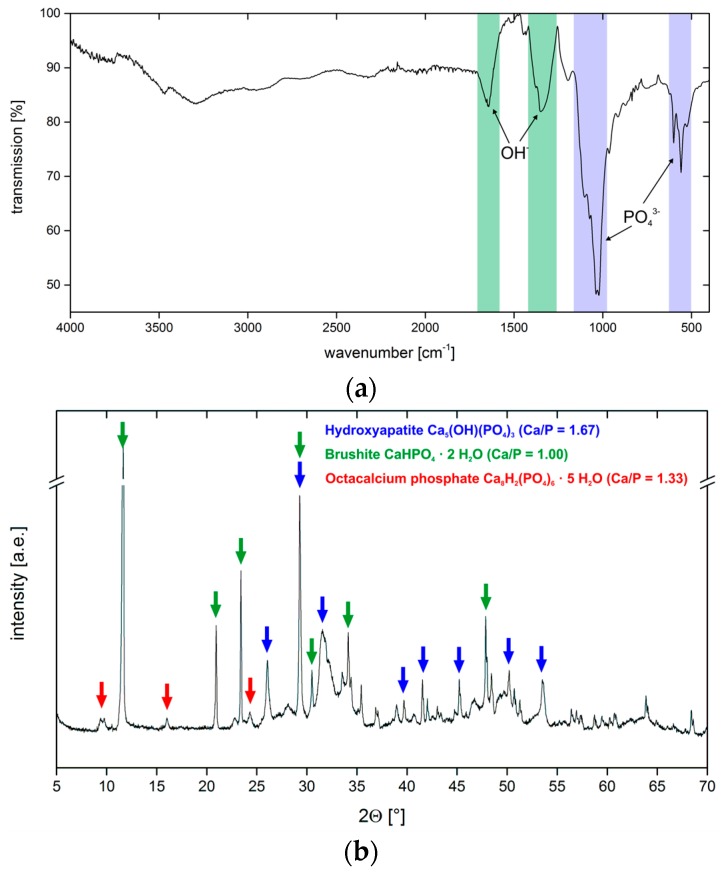
(**a**) Representative IR spectrum of the sample G01 (see Table 3 for sample designations). The other IR spectra are essentially identical with varying intensities of individual bands. (**b**) Representative XRD pattern of the sample G06. The other diffractograms are essentially identical with varying intensities of the individual reflections.

IR spectra (Figure 7a) show bands that are characteristic for phosphate, carbonate, and hydroxyl groups. Broad bands at 3467 and 3292 cm^−1^ are assigned to hydroxyl groups as are the medium intensity bands at 1649 and 1348 cm^−1^. Weak bands at 1446 and 872 cm^−1^ are due to the presence of carbonate ions. A strong doublet at 1038 and 1022 cm^−1^ (ν_3_ PO_4_^3−^) and a strong triplet at 600, 559, and 526 cm^−1^ (ν_4_ PO_4_^3−^) stems from phosphate and hydrogen phosphate ions [20,21].

Figure 7b shows the corresponding X-ray diffraction (XRD) data. The XRD patterns are rather complex and show reflections that can be assigned to HAP (ICDD 98-000-1706) at 26.1° (002), 29.3° (210), 32° (112), 34.1° (202), 39.7° (212/310), 43.4° (113), 46.7° (222), 50.2° (213), and 53.5° (004) 2θ. Further reflections are due to the presence of brushite (ICDD 98-017-2261); these reflections are observed at 11.7° (020), 20.9° (021), 23.4° (040), 29.3° (041), 30.5° (−221), and 34.4° (−220) 2θ. Finally, the presence of OCP (ICDD 98-006-5347) is confirmed by reflections at 9.4° (010), 9.8° (011), 16.1° (110), and 24.3° (12-1) 2θ.

**Figure A2:**
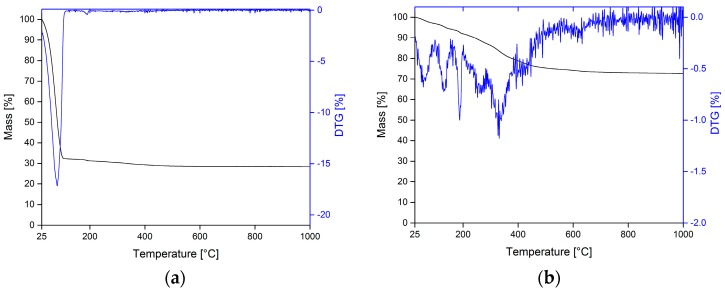
Representative TGA and DTG curves of: a wet sample (G01) (**a**); and a dry sample (G01) (**b**).

Thermogravimetric analysis (TGA) of a wet sample (Figure A2a, Appendix A) shows one single mass loss signal essentially starting at room temperature and ranging to ca. 105 °C with a peak maximum in differential thermogravimetric analysis (DTG) around 86 °C. The corresponding mass loss is 69%, presumably water. An additional, less pronounced mass loss of ca. 3% is visible between 150 and 500 °C; this weight loss is likely due to degradation of the hydrogel. No further mass loss can be observed up to 1000 °C. The residual mass of 28.5% is assigned to inorganic material, mainly calcium phosphate and pyrophosphate. 

In contrast to the wet samples, all dry samples (Figure A2b, Appendix A) show a much less clearly defined weight loss of ca. 15%, likely due to water desorption, between room temperature and 300 °C. Between 300 and 400 °C, the slope of the TGA curve appears slightly steeper, although this is difficult to quantify. At ca. 450 °C, a plateau is reached and there is essentially no further weight loss until a residual mass of 73%, presumably calcium phosphate, is reached at 1000 °C. 

As stated above, chitosan was also evaluated for hydrogel layer formation. However, compared to the gelatin solutions described above, the chitosan concentrations in the Ca(NO_3_)_2_ solution had to be reduced to favor CP deposition; concentrations analogous to those used for the gelatin-based systems lead to complete or near-complete inhibition of CP formation. 

The NaOH treatment of the PLA scaffolds (see experimental parts and Figure 2) leads to the formation of carboxylic acids and carboxylate groups on the PLA surface. We therefore initially rationalized that the electrostatic interaction between the negatively charged carboxylates on the PLA surface and the positively charged ammonium groups of the chitosan would provide a strong attachment of the chitosan/CP layer to the PLA. As a result, initial experiments were done without previous silanization. Indeed, thick mineral films form during the 15-cycle deposition treatment (Figure 2, Table 5) and all scaffolds are covered with a white layer. However, the adhesion of the coating to the underlying PLA is poor. Although no mineral directly falls off the PLA scaffold, it is easy to scrape off some material with a spatula. As a result, the chitosan-based films were also made via the process established for the gelatin-based films described above (Figure 2). 

**Figure 8:**
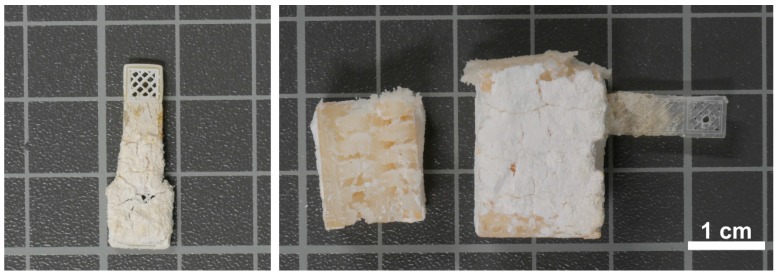
Representative photographs of mineral coated samples of type: P2 sample (C13) (**left**); and (sample C19) P3 (**right**).

Silanization before mineralization leads to better adhesion between the hydrogel/CP hybrid films and the PLA scaffold. The results are, with the exception of the lower chitosan contents in the films, comparable to the gelatin-based materials. As described for the gelatin-based samples, the concentrations of Ca(NO_3_)_2_, Na_2_HPO_4_, chitosan, and GA directly influence both the amount of deposited CP and the homogeneity of the coating. Salt concentrations below 0.05 mol∙L^−1^ lead to no significant CP deposition while concentrations over 0.3 mol∙L^−1^ lead to very rapid mineralization producing inhomogeneous surface coatings. Similar to gelatin, the chitosan concentration influences the CP mineralization. Concentrations over 0.5% completely inhibit CP deposition. Overall, these experiments show that the best conditions for the formation of a homogeneous chitosan/CP hybrid coating is the condition used for sample C12, Table 6. As a result, P3 scaffolds were coated using this optimized protocol showing that true 3D scaffolds can indeed be coated using the same experimental conditions (Figure 8).

After mineralization of the silanized samples, they show (Figure 8) a thick white coating covering the entire scaffold. The chitosan/CP hybrid layer is firmly connected to the scaffold and the open pores of the P1 and P2 scaffolds are not visible anymore as they are densely covered with the mineral layer. The true 3D scaffolds (P3) are coated with a thick mineral layer on the outside. The layer has again a thickness of about 0.5 mm. This is evident after cutting some samples open. All samples show a few cracks, likely due to the drying process, but the coating is firmly attached to the scaffold. It can be scratched off with a spatula producing smaller pieces; powders of the material can only be obtained by grinding in a mortar. 

**Figure 9:**
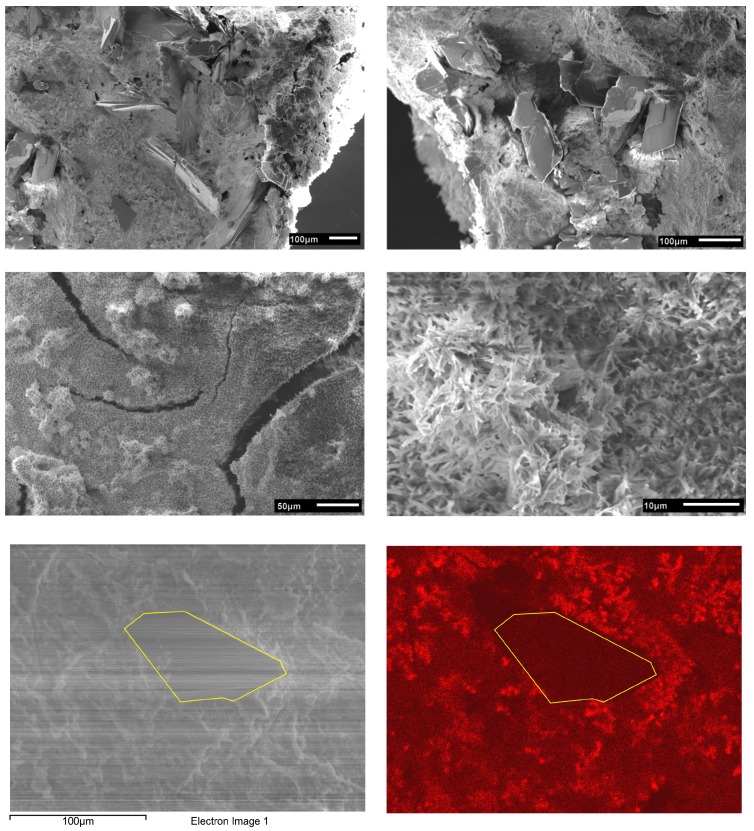
Representative SEM images of a chitosan/CP composite surface (sample C12). All images are from the same sample but taken at different locations and different magnifications to show the morphological and dimensional diversity. Bottom images show an Na elemental map in sample C12 and the corresponding SEM image. The plates are essentially sodium-free and the surrounding area shows a sodium content of ca. 5 atom %.

SEM (Figure 9) shows that the mineral/chitosan layers are quite similar to the samples described above for the gelatin-based materials. All samples show a mixture of plate-like crystals with dimensions on the order of 50 to 200 µm that are embedded in a matrix of needle-like or spherical particles with no apparent order. While the plates are present throughout the sample, they are not distributed evenly; rather there are regions where they appear as large clusters and adjacent regions where only a few plates exist. 

In contrast to the plates, which have a rather well-defined morphology, the needles are strongly interconnected and present as densely packed aggregates and intertwined architectures. Lower magnification images (Figure 9 middle left) show that the needles form large “carpets” of intertwined and closely connected needles. As stated above, the large plates are likely DCPD while the needles and smaller particles are likely OCP and HAP.

The EDXS data (Table 7) resemble the data obtained from the gelatin-based composites. Again the plate-like features show Ca/P ratios of ca. 0.9, which again supports the notion that these plates are DCPD (Ca/P = 1). Both the disordered areas without large plates and the interconnected needles show a very similar composition with a Ca/P ratio of around 1.5 and approximately 5% of Na. Further experiments using the EDXS mapping mode clearly show that only the needles and spherical particles contain Na while the plates do not, Figure 9.

**Figure 10:**
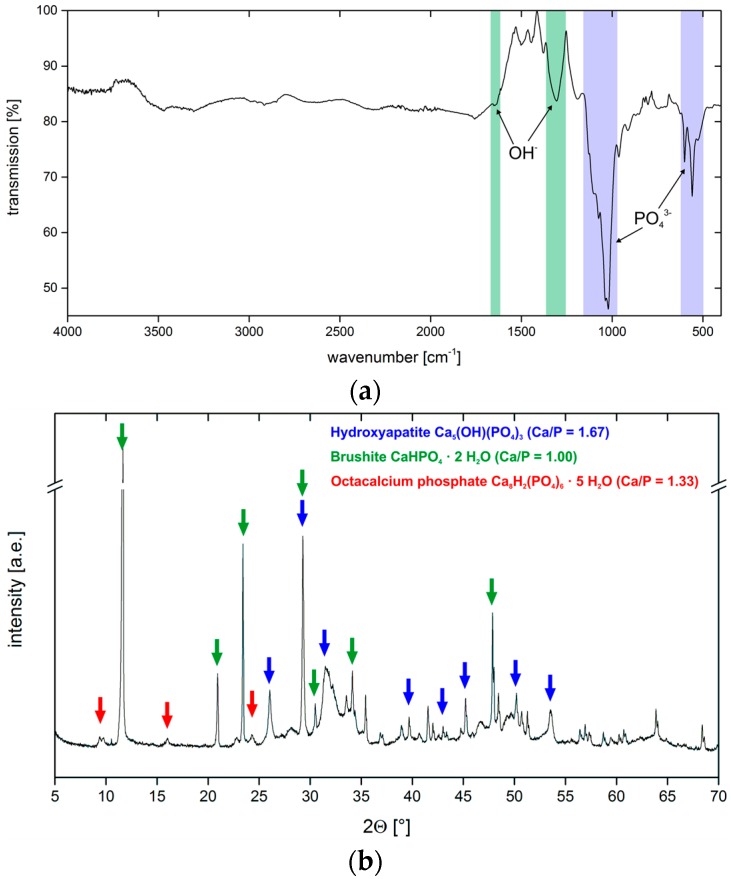
(**a**) IR spectrum obtained from sample C12. The other IR spectra are essentially identical with varying intensities of individual bands. (**b**) X-ray diffractogram of sample C20. The other diffractograms are essentially identical with varying intensities of individual reflections.

IR spectra (Figure 10a) of the coatings show weak broad bands at 3471, 3305, and 1649 cm^−1^ and a sharp medium-intensity band at 1306 cm^−1^; these bands are indicative of hydroxyl groups from hydroxyapatite and chitosan. Weak bands at 1444 and 870 cm^−1^ (ν_3_ PO_4_^3−^) are characteristic for carbonate ions. A sharp double band at 1038 and 1022 cm^−1^ (ν_4_ PO_4_^3−^) and a sharp triple band at 602, 559, and 528 cm^−1^ are due to phosphate and hydrogen phosphate ions [20,21].

XRD (Figure 10b) confirms the presence of several crystal phases. All XRD patterns are again rather complex and show reflections that can be assigned to HAP (ICDD 98-000-1706); these reflections can be found at 26.1° (002), 29.3° (210), 31.5° (112), 34.1° (202), 39.7° (212/310), 43.4° (113), 46.7° (222), 50.2° (213), and 53.6° (004) 2θ. Additional brushite (ICDD 98-017-2261) reflections can be identified at 11.7 (020), 20.9° (021), 23.4° (040), 29.3° (041), 30.5° (−221), and 34.4° (−220) 2θ. Finally, the remaining reflections at 9.4° (010), 9.8° (011), 16.0° (110), 24.3° (12-1) 2θ can be assigned to OCP (ICDD 98-006-5347).

**Figure A3:**
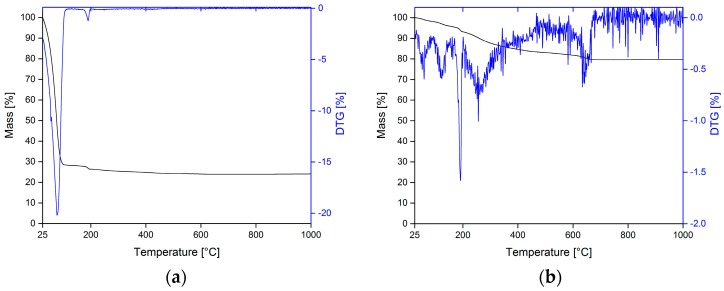
TGA and DTG curve from: a wet sample (C12) (**a**); and a dry sample (C12) (**b**).

Similar to the experiments described for the gelatin-based composites, TGA and DTG investigations were performed for the chitosan-based coatings on wet and dry samples (Figure A3a, Appendix A). The wet sample shows a rather large mass loss of 72% between room temperature and ca. 105 °C; the corresponding DTG peak maximum is around 78 °C. A second smaller mass loss of ca. 2% is observed between 170 and 205 °C; this signal has a maximum in the DTG data at ca. 190 °C. A less pronounced mass loss of ca. 2% is visible between 205 and 600 °C. The residual mass at 1000 °C is ca. 24%; presumably this is calcium phosphate and pyrophosphate. 

The dry sample (Figure A3b, Appendix A) shows—similar to the gelatin-based composite—a less clearly defined weight loss of ca. 20% between room temperature and 670 °C. Similar to the wet sample, there is a DTG peak at ca. 190 °C with approximately the same intensity of −1.5%. The residual mass of 80% at 1000 °C is again calcium phosphate and pyrophosphate.

## 4. Discussion

This work uses 3D printing to generate PLA scaffolds, which, after modification with a suitable surface layer, can be coated with a thick CP/hydrogel hybrid layer. As the 3D printed object can be made to match a specific defect in, for example, a bone, the process described here offers a suitable and highly versatile pathway towards personalized implants or towards objects like implantable and biodegradable materials with a defined shape, surface chemistry, and architecture. The process is scalable, highly adaptable, simple, and cheap and thus lends itself to applications in the biomaterials field.

First experiments (Table 1), however, show that pure CP only poorly adheres to the PLA surface. Moreover, the CP layer itself is not mechanically stable and easily disintegrates. Consequently, a surface modification protocol was successfully established: prior to surface coating and mineralization, the 3D printed PLA scaffold was surface-hydrolyzed with NaOH to generate hydroxyl and carboxyl groups on the PLA surface; these act as reactive centers for the subsequent scaffold modification. The second step is the modification of the surface with APTES. The dangling amino groups from the APTES modification on the PLA surface enable the efficient binding of either gelatin or chitosan on the surface via glutaraldehyde crosslinking through the formation of imine bonds [18]. The concurrent formation of the polymer surface layers with CP deposition provides access to a homogeneous polymer/CP hybrid coating layer on the PLA (Figure 2).

The success of the NaOH treatment is evidenced via contact angle measurements. The CA of pure PLA after the printing process is around 78°, consistent with literature [18,22]. According to literature, however, NaOH treatment should lead to a significant reduction of the CA [22]. In contrast to these data, the current work finds a significant CA increase with NaOH treatment. We assign this to a pronounced roughening of the surface as evidenced by AFM and qualitatively also by SEM data (Figure 3). As the CA depends on both the chemical and the physical properties of a surface [23,24], there are likely two opposing effects: while the NaOH treatment cleaves PLA ester bonds (which produce hydroxyl and carboxylic acid groups on the surface favoring a lower contact angle) the increased roughness leads to a more hydrophobic surface. Apparently, here, the second effect prevails and this leads to a more hydrophobic surface. 

The subsequent silanization reaction is the somewhat more difficult to prove; the silane layer is very thin and consequently, IR spectra show no indication of Si–O vibrations as reported in the literature [25,26]. Moreover, there is no indication of N–H vibrations in IR spectroscopy (Figure 7 and Figure 9); this is because the signal overlaps with O–H vibrations from the (cleaved) PLA [2]. Moreover, there is no significant change in the contact angle. 

EDXS however provides clear evidence of Si showing that a silica/SiO_x_ layer is present, Figure 4. The detection of nitrogen in EDXS is more difficult. This is because the N K_α_ emission is at 0.4 keV, close to the O K_α_ signal. Indeed, Figure 4 shows a shoulder next to the oxygen emission peak; this signal may be due to nitrogen but the low intensity prevents a clear assignment. A similar observation has also been made by Lin et al. [27] on CP coated PLA fibers: although nitrogen was clearly present in these samples, EDXS barely showed an N emission peak. Moreover, as stated in the Results Section, the total concentration of N in the current samples is many orders of magnitudes lower than the O concentration and hence the nitrogen signal must be much weaker than the oxygen signal. 

The last step, the formation of the hybrid CP hydrogels from gelatin or chitosan with glutaraldehyde, is described in the literature [28,29,30] and we have found these protocols to be reliable and reproducible. Gelatin and chitosan were chosen because of their biocompatibility and their availability. While gelatin is similar to collagen, the organic main component of bone [31,32], chitosan is interesting for its antibacterial activity. Possibly a combination of the two polymers in one coating could thus be beneficial for both mineral formation and protection from infections of an implant site. This aspect is however beyond the scope of this article and will be addressed in the future. 

The key novelty of the work presented here is the direct combination of hydrogel deposition and mineral layer deposition: every dip coating cycle produces a layer of polymer and at the same time a layer of mineral on the PLA scaffold surface. This is interesting from several points of view: (1) the deposition process is simplified as there are less subsequent steps until the final material is obtained; (2) the hybrid film is very homogeneous because—at the same time—both the mineralization matrix and the mineral are deposited; and (3) the growing coating is constantly stabilized by the repeated application of glutaraldehyde in every deposition cycle. 

Despite these advantages, detailed investigations show that the process of hydrogel formation is parameter-sensitive (Table 5). For example, high polymer concentrations lead to a complete inhibition of CP mineralization. Furthermore, different pH values in the two dip coating solutions lead to different calcium phosphate phases. The Ca(NO_3_)_2_/gelatin or Ca(NO_3_)_2_/chitosan solutions have a pH of ca. 5 while the Na_2_HPO_4_/GA solutions have a pH of ca. 8. As the pH strongly affects the phase selection in CP mineralization [8,11], different (crystal) phases can be expected and indeed, a mixture of CP phases is observed from the alternating dip coating process. In fact, the observed mixture of DCPD, OCP, and HAP is quite attractive for implants, as all CP phases have different stabilities and degradation rates in physiological conditions. This will lead to an interesting and potentially useful dissolution profile of the coating because the CP layer will not dissolve at once but rather over an extended period. Such a stability profile could offer an interesting time window for bone cells such as osteoblasts and osteoclasts to remodel the materials surface. 

Moreover, the current samples, in particular the P3 scaffolds, show that the pores of the scaffolds are so coated with mineral that the inner surfaces are essentially not coated. This is an issue when considering a real implant because some of the implant surface will then be bare PLA. However, designing a P3-like scaffold with much larger pores will open up space for mineralization of the scaffold interior; in particular when combined with a lower number of deposition cycles. Both parameters (deposition cycles and details of the scaffold architecture) are easily implemented in a modified version of the protocol. Therefore, the protocol described here are only the basis for a highly attractive and very flexible approach towards a large library of new hybrid biomaterials. Overall, our approach to the rather complex materials reported here is simple, versatile, and lends itself to applications in personalized medicine. Both materials are quite similar and should have their advantages: while chitosan may add a certain anti-infection property, gelatin may be attractive for bone ingrowth. To evaluate which material is more promising in a final application, both mechanical properties and biological properties will have to be evaluated. 

## 5. Conclusions

3D printing of PLA is well established, but the design of suitable materials for the biomaterials field is still a challenge. The approach reported here has several advantages over state of the art technology: it is versatile, scalable, and cheap. Moreover, using for example microtomography information, a scaffold can be specifically tailored for a specific defect; the subsequent mineralization process will render the 3D printed scaffold biocompatible and the combination of materials will also favor degradation over time. In summary, the approach provides access to personalized implant materials with a biocompatible and possibly osteoinductive coating in a simple, scalable, and highly adaptable process. Clearly, the biocompatibility of these new materials will need to be investigated and some open questions remain (replacement of APTES and GA, use of other polymers, mechanical properties of the materials, biocompatibility, and degradability profiles) but the approach is highly promising because of its simplicity, adaptability, and versatility.

## Figures and Tables

**Table 1:** Reference (Rxx) samples without PLA modification prior to coating with the mineral layer.

Sample Number	Scaffold Type	Solution 1	Solution 2	Weight Change
R01	P2	0.10 M Ca(NO_3_)_2_	0.10 M Na_2_HPO_4_	+0.0117 g (+27.9%)
R02	P2	0.20 M Ca(NO_3_)_2_	0.20 M Na_2_HPO_4_	+0.0327 g (+81.8%)

**Table 2:** Effects of NaOH treatment on PLA scaffolds.

Parameter	Before Hydrolysis	After Hydrolysis
Weight of Type P2 [g]	0.0863 ± 0.0019	0.0782 ± 0.0019 (−(9 ± 1)%)
Weight of Type P3 [g]	2.575 ± 0.008	2.427 ± 0.032 (−(6 ± 2)%)
Contact angle [°]	78.0 ± 17.7	102.9 ± 9.2

**Table 3:** Mineralization parameters for reactions with gelatin (Gxx) and glutaraldehyde. Mineralization protocols leading to the most homogeneous coatings are highlighted in bold. G6 and G7 are identical to G1 and demonstrate the reproducibility.

Sample Number	Scaffold Type	Solution 1	Solution 2	Weight Change
**G01**	**P2 ^a^**	**0.20 M Ca(NO_3_)_2_** **+ 1.0% gelatin**	**0.20 M Na_2_HPO_4_** **+ 0.5% GA**	**+0.0483 g** **(+57.4%)**
G02	P2 ^a^	0.20 M Ca(NO_3_)_2_ + 0.2% gelatin	0.20 M Na_2_HPO_4_ + 0.5% GA	+0.0522 g (+62.0%)
G03	P2 ^a^	0.20 M Ca(NO_3_)_2_ + 0.1% gelatin	0.20 M Na_2_HPO_4_ + 0.5% GA	+0.0542 g (+64.8%)
G04	P2 ^a^	0.30 M Ca(NO_3_)_2_ + 0.2% gelatin	0.30 M Na_2_HPO_4_ + 0.1% GA	+0.1043 g (+125.7%)
G05	P2 ^a^	0.20 M Ca(NO_3_)_2_ + 0.2% gelatin	0.20 M Na_2_HPO_4_ + 0.1% GA	+0.0526 g (+60.7%)
**G06**	**P2 ^b^**	**0.20 M Ca(NO_3_)_2_** **+ 1.0% gelatin**	**0.20 M Na_2_HPO_4_** **+ 0.5% GA**	**+0.0571 g** **(+72.9%)**
**G07**	**P3 ^b^**	**0.20 M Ca(NO_3_)_2_** **+ 1.0% gelatin**	**0.20 M Na_2_HPO_4_** **+ 0.5% GA**	**+0.4052 g** **(+16.7%)**

^a^ Silanization with 1% APTES in toluene; ^b^ silanization with 10% APTES in EtOH prior to hydrogel formation/mineralization.

**Table 4:** EDXS data of sample G01. Note that quantification of EDXS data for these materials is a challenge and that the values reported here have been obtained using an internal standard procedure.

Element	Plates [atom %]	Disordered Area [atom %]
O	67.81	49.72
Na	0.00	1.54
P	16.39	20.16
Ca	15.81	28.58
Ca/P	0.96	1.42

**Table 5:** Mineralization parameters for reactions with chitosan (samples named Cxx) and glutaraldehyde without previous silanization.

Sample Number	Scaffold Type	Solution 1	Solution 2	Weight Change
C01	P1	0.10 M Ca(NO_3_)_2_ + 1.0% chitosan	0.10 M Na_2_HPO_4_ + 1.0% GA	- ^c^
C02	P1	0.10 M Ca(NO_3_)_2_ + 0.1% chitosan	0.10 M Na_2_HPO_4_ + 1.0% GA	+0.0036 g (+11.2%)
C03	P1	0.10 M Ca(NO_3_)_2_ + 0.1% chitosan	0.10 M Na_2_HPO_4_ + 1.0% GA	+0.0064 g (+18.4%)
C04	P1	0.20 M Ca(NO_3_)_2_ + 0.1% chitosan	0.20 M Na_2_HPO_4_ + 1.0% GA	+0.0358 g (+118.9%)
C05	P1	0.20 M Ca(NO_3_)_2_ + 0.1% chitosan	0.20 M Na_2_HPO_4_ + 0.1% GA	+0.0358 g (+118.9%)
C06	P2	0.20 M Ca(NO_3_)_2_ + 1.0% chitosan	0.20 M Na_2_HPO_4_ + 1.0% GA	- ^c^
C07	P2	0.20 M Ca(NO_3_)_2_ + 1.0% chitosan	0.20 M Na_2_HPO_4_ + 0.1% GA	- ^c^

^c^ Too high chitosan concentration inhibits the mineralization process.

**Table 6:** Mineralization with chitosan and glutaraldehyde with previous silanization. Mineralization protocols leading to the most homogeneous mineral layer deposition are highlighted in bold.

Sample Number	Scaffold Type	Solution 1	Solution 2	Weight Change
C10	P2 ^a^	0.10 M Ca(NO_3_)_2_ + 0.1% chitosan	0.10 M Na_2_HPO_4_ + 1.0% GA	+0.0311 g (+16.9%)
C11	P2 ^a^	0.15 M Ca(NO_3_)_2_ + 0.2% chitosan	0.15 M Na_2_HPO_4_ + 0.5% GA	+0.0232 g (+27.8%)
C12	P2 ^a^	0.20 M Ca(NO_3_)_2_ + 0.1% chitosan	0.20 M Na_2_HPO_4_ + 0.1% GA	+0.0372 g (+44.4%)
C13	P2 ^a^	0.20 M Ca(NO_3_)_2_ + 0.1% chitosan	0.20 M Na_2_HPO_4_ + 1.0% GA	+0.0428 g (+52.5%)
C14	P2 ^a^	0.30 M Ca(NO_3_)_2_ + 0.1% chitosan	0.30 M Na_2_HPO_4_ + 1.0% GA	+0.1180 g (+146.0%)
C15	P2 ^a^	0.20 M Ca(NO_3_)_2_ + 0.2% chitosan	0.20 M Na_2_HPO_4_ + 1.0% GA	+0.0508 g (+62.5%)
C16	P2 ^a^	0.15 M Ca(NO_3_)_2_ + 0.1% chitosan	0.15 M Na_2_HPO_4_ + 1.0% GA	+0.0199 g (+24.4%)
C17	P2 ^a^	0.15 M Ca(NO_3_)_2_ + 0.2% chitosan	0.10 M Na_2_HPO_4_ + 1.0% GA	+0.0237 g (+28.5%)
C18	P2 ^a^	0.15 M Ca(NO_3_)_2_ + 0.1% chitosan	0.15 M Na_2_HPO_4_ + 0.5% GA	+0.0254 g (+30.4%)
**C19**	**P3 ^b^**	**0.20 M Ca(NO_3_)_2_** **+ 0.1% chitosan**	**0.20 M Na_2_HPO_4_** **+ 0.1% GA**	**+0.3973 g** **(+16.2%)**
**C20**	**P2 ^b^**	**0.20 M Ca(NO_3_)_2_** **+ 0.1% chitosan**	**0.20 M Na_2_HPO_4_** **+ 0.1% GA**	**+0.0343 g** **(+44.3%)**

^a^ Silanization with 1% APTES in toluene; ^b^ silanization with 10% APTES in EtOH.

**Table 7:** EDXS data of chitosan-calcium phosphate-composite sample C12. Note that quantification of EDXS data from these materials is a challenge and that the values reported here have been obtained using an internal standard procedure.

Element	Plates [atom %]	Disordered Area [atom %]
O	67.75	56.30
Na	0.00	5.26
P	17.43	15.17
Ca	14.82	23.26
Ca/P	0.85	1.53
